# Understanding how patients perceive physician wellness and its links to patient care: A qualitative study

**DOI:** 10.1371/journal.pone.0196888

**Published:** 2018-05-15

**Authors:** Jane B. Lemaire, Darby Ewashina, Alicia J. Polachek, Jaya Dixit, Verna Yiu

**Affiliations:** 1 Cumming School of Medicine, Department of Medicine, University of Calgary, Calgary, Alberta, Canada; 2 W21C Research and Innovation Center, Cumming School of Medicine, University of Calgary, Calgary, Alberta, Canada; 3 Cumming School of Medicine, Department of Psychiatry, University of Calgary, Calgary, Alberta, Canada; 4 University of Calgary, Calgary, Alberta, Canada; 5 Alberta Health Services, Edmonton, Alberta, Canada; Hong Kong Polytechnic University, HONG KONG

## Abstract

Despite increased interest in physician wellness, little is known about patients’ views on the topic. We explore patients’ perceptions of physician wellness and how it links to patient care. This exploratory, qualitative study employed semi-structured interviews with a convenience sample of 20 patients from outpatient care settings in a western Canadian city. Using inductive thematic analysis, interview transcripts were independently coded by two authors and then discussed to ensure consensus and to abstract into higher-level themes. Three overarching premises were identified. First, patients notice cues that they interpret as signs of physician wellness. These include overt indicators, such as a physician’s demeanor or physical appearance, along with a general impression about a physician’s wellness. Second, patients form judgments based on what they notice, and these judgments affect patients’ views about their care; feelings, such as trust, in their interactions with physicians; and actions, such as following care plans. Third, participants perceive a bi-directional link between physician wellness and patient care. Physician wellness impacts patient care, but physician wellness is also impacted by the care they provide and the challenges they face within the healthcare system. Patients’ judgments regarding physician wellness may have important impacts on the doctor-patient relationship. Furthermore, patients appear to have a nuanced understanding about how physicians’ work may put physicians at risk for being unwell. Patients may be powerful allies in supporting physician wellness initiatives focused on the shared responsibility of individual physicians, the medical profession, and healthcare organizations.

## Introduction

Physician wellness is an important topic that has received increasing attention over recent years. Physicians are at high risk for serious health consequences including burnout [1–3], substance abuse [4, 5], depression, and suicide [6–9]. Physician wellness also impacts healthcare delivery, such that physician stress, burnout, and job dissatisfaction may harm doctor-patient interactions and result in suboptimal patient care [10–14]. Furthermore, physicians’ health behaviors can influence how they counsel patients [15–17], such that a positive relationship exists between physicians’ and patients’ preventative health practices such as mammography, colorectal cancer screening, and vaccinations [18]. Patients may value being privy to the behaviors and health practices of physicians in addition to receiving their knowledge and expertise, implying that physicians have a powerful public health influence by setting an example for patients. With mounting evidence regarding the prevalence and impacts of unwell physicians, physician wellness was proposed as a missing quality indicator for healthcare systems [19]. There is also advocacy for adding a Fourth Aim—healthy work-life for healthcare providers—to the Triple Aim of health care reform, which currently focuses on improving patient experience and population health while controlling costs [20].

Despite this importance of physician wellness, physicians sometimes feel that striving for wellness is at odds with their professionalism and that patient care should come above all else. For example, many physicians report working when ill [21], and some have identified beliefs that behaviors such as eating at work are unprofessional and may be considered so by patients [22, 23]. While the medical profession may hold this view, it is important to also consider patients’ perceptions of physician wellness and how it links to patient care, especially given the increased emphasis on patient-centered care. To date, however, little research has explored this viewpoint. Previous research on patients’ perspectives has focused on physician body weight, with Puhl’s work demonstrating that patients may hold weight biases such that patients of normal weight may be less trusting of and less likely to follow medical advice from overweight physicians [24]. Participants in Puhl’s study also reported being more likely to change providers if a physician was overweight.

Although understanding patients’ perceptions of physician weight is important, a broader grasp of patients’ views about physician wellness is needed, in particular to explore if these views are linked to feelings about their care. This understanding will help in better conceptualizing physician wellness and understanding the impact that perceived physician wellness has on doctor-patient interactions. This study therefore explores the following research question: What are patients’ perspectives on physician wellness and how physician wellness links to patient care?

## Methods

### Study design

This qualitative study uses a constructivist orientation to understand patients’ perceptions. This approach honors subjective meanings, recognizing that patients may hold diverse views of physician wellness as a result of varied experiences and interactions [25, 26]. Our team included a physician with expertise in physician wellness, a physician in a high-level leadership role, a physician trainee with an interest in physician wellness, and three social scientists with master’s level training in sociology and expertise in physician wellness and qualitative research methods. There were no pre-existing relationships with participants.

### Participant recruitment

Patients over the age of 18 were recruited from outpatient settings in Calgary, Alberta, Canada, between December 2014 and November 2015 using a non-probability, convenience sampling strategy. To ensure participants had exposure to a broad range of healthcare services, recruitment posters were displayed at an urgent care center that also houses various primary care clinics, an outpatient diagnostic and treatment center, and all locations of a city-wide laboratory collection service. Interested patients who contacted the researchers were given information about the study and invited to participate in a telephone interview. Researchers also staffed a study information booth at the urgent care center on three occasions. Patients approaching the booth were given information about the study and invited to participate in an interview on-site or by telephone at a later date. Participants were told the researchers were interested in exploring how patients perceive physician wellness and how it links to patient care. Physician wellness was not defined for participants, as we sought to understand how physician health and well-being is perceived by patients. Potential participants were told the results could be used to help physicians realize the professional importance of caring for their wellness. Given the voluntary sampling strategy, no one refused to participate. No participants withdrew.

### Data collection

The physician trainee (DE) and two social scientists (AP and one other) working as research associates at the University of Calgary conducted 20 one-on-one interviews between March and November 2015. This number was deemed adequate to achieve theoretical saturation where no new themes emerged, without being too large for detailed analysis [27,28]. We interviewed 11 (55%) females and nine (45%) males, with an average age of 57.2 years (range = 25 to 92 years). Seven (35%) were married, two (10%) lived with a common-law partner, four (20%) were divorced, three (15%) were widowed, and four (20%) were single/never married. Most participants (75%) had children. Eight (40%) participants had completed a college or university education, ten (50%) had attended some college or university courses, and two (10%) had no post-secondary education beyond high school. Compared to others their age, one (5%) participant rated their physical health as excellent, six (30%) as very good, eight (40%) as good, two (10%) as fair, and three (15%) as poor. Compared to others their age, three (15%) participants rated their mental or emotional health as excellent, six (30%) as very good, seven (35%) as good, three (15%) as fair, and one (5%) was unreported. Four (20%) participants interacted with the healthcare system every week, nine (45%) almost every month, four (20%) every two or three months, and three (15%) less than once a year. Ten (50%) participants were retired or semi-retired, four (20%) were unemployed, and six (30%) were employed.

All interviewers were female. Thirteen interviews were conducted by telephone and seven in person at the urgent care center study information booth. Interviews lasted an average of 46.4 minutes (range = 20.4 to 104.4 minutes). All interviews were digitally recorded and explicit verbal consent was obtained prior to proceeding. A semi-structured interview guide was used to explore standardized questions with all participants, while also allowing for unique prompts to further explore new ideas raised by participants (see S1 File). The interview guide was developed collaboratively by the research team, including physician members to ensure the questions resonated with physicians.

Participants were encouraged to draw on personal experiences, examples from friends or family, and general perceptions, but were not asked to evaluate specific physicians. This ensured that participants could draw on a variety of examples and describe their perceptions of physician wellness more broadly, not just with regard to specific providers. Interviews were transcribed verbatim by the physician trainee (DE) and a research assistant not involved in data collection or analysis. Transcripts were identified using a unique identification number. Participant names linked to identification numbers were only seen by the three interviewers and filed in a separate secure location. Excerpts presented here are de-identified. The study was approved by the University of Calgary’s Conjoint Health Research Ethics Board.

### Data analysis

Inductive thematic analysis began after data collection was completed. Several measures were taken to increase the trustworthiness and confirmability of our results. The physician trainee (DE) and one social scientist (AP) independently read each interview transcript, notated initial codes without a-priori categorization, and then met to compare findings and achieve consensus through discussion. Data were managed using NVivo 10 (QSR International, Doncaster, Australia). Once all interviews were coded, these two analysts iteratively grouped similar codes into themes. Similar themes were then grouped into larger overarching themes. A final interpretation of the themes generated three overarching premises regarding how patients may perceive physician wellness and its link to patient care. Study results were then discussed by all authors to further develop theoretical concepts and identify relationships between themes. The team’s diverse backgrounds (i.e., practicing physician researcher, physician in a leadership role, sociology trained social scientists, physician trainee), all with an interest in physician wellness, informed the results and reduced bias by incorporating different positions and perspectives. Thematic saturation was reached, where no new themes emerged [26–28]. Results were not subsequently checked with participants.

## Results

Three overarching premises emerged regarding how patients perceive physician wellness and its links to patient care. First, patients notice cues in physicians that they interpret as signs of wellness or unwellness. Second, patients form judgments based on what they notice, and these judgments impact how they view their care, feel about the encounter, and act within the doctor-patient interaction. Third, patients perceive a bi-directional link between physician wellness and patient care. Physician wellness impacts patient care, but physician wellness is also impacted by the care they provide to patients.

The first premise is that, while it may be difficult to discern physician wellness, most patients notice cues about physicians that they *interpret* as signs of wellness or unwellness (see Table 1). These cues may be easily observable such as physical appearance, demeanor, work pace, and signs of stress, or may be a more general impression of physician wellness. For example, participants described the ability to “sense” an intangible energy, particularly when the doctor-patient relationship is established over time, where patients can observe changes in a physician’s emotional stability, weight, or demeanor between visits.

**Table 1:** What patients notice about physicians’ wellness.

Themes	Sub Themes	Description	Sample Quotations ^a^
Overt Indicators	Physical Appearance	How the doctor looks e.g., age, gender, weight, appearing sick, and being unclean or unkempt	*“You look for the physical indicators of their body type*, *certainly you can see wear on the face…how they’re composed and put themselves together…the way they dress*, *the way they maintain their clothes throughout the day…”* [10]
	Demeanor	How the doctor acts e.g., tone of voice, attitude, behaviors, being confident, arrogant, anxious	*“Agitation*, *um distractibility…you could potentially feel like they’re closed off and that something’s bothering them…And um*, *not willing to talk I guess*. *Like they’re uncomfortable or you get the sense that they’re more comfortable working with someone in an operating table than with dealing with you*.*”* [4]
	Work Pace	How much time the doctor seems to have for patients e.g., physical pace of movement, trying to do more than time allows, rushing patients through	*“…I had to ask to bring him back in again because all the [skin] tags that we had agreed to take off…he missed half of them the first time*, *and a third of them the second time*, *after I called him in the third time*, *he actually completed the job*, *so for me that was a…guy that was sort of flying around…with a bunch of patients in different rooms…His coat was kind of flying behind him*.*”* [3]
	Signs of Stress	How tense or pressured the doctor appears to be e.g., lacking cognitive focus, looking overwhelmed, busy, or overworked	*“…they are looking…totally run off their feet and totally stressed out and… like you know you are the fifty-sixth thousandth patient that they have seen and they can’t keep the case straight*, *they can’t keep your record straight*, *they can’t keep your question straight*, *it kind of leads you to wonder is he in any condition to be doing this right now you know*.*”* [18]
General Impression	A Sense	An intangible or non-verbal energy that patients perceive from doctors	*“Patients may have a sixth sense of a doctor’s wellness or unwellness*.*”* [7] *“If a person is in good health*, *something emanates from them*.*”* [11]
	Consistency between Visits	How much change there is in the doctor between visits e.g., emotional stability, ability to convey a sense of equanimity, change in weight or demeanor	*“…this physician…started off…fairly trim [but by the end] she must have put on an extra hundred pounds and it just made me think that she wasn’t eating well…and physically she just didn’t look well and I don’t know if that was just because of the weight or because…she was more relaxed during the start and towards the end she just seemed more tense all the time I guess…She always looked very tired and stressed out I guess at the end*.*”* [13]
	Job Fulfillment	How much the doctor appears to enjoy his/her job e.g., use of humor, appearing upbeat and happy, enthusiastic about work	*“I tried to ask my family doctor…to never send me to that [specialist] again*, *because he looked like he was about ready to put a bullet in his head*, *he hated his job so much*.*”* [3]

^a^ Numbers indicate the source of the quote based on the participant’s unique identifier.

The second premise is that patients form judgments based on what they notice about physicians, and these judgments may impact patients (see Table 2). Patients who judge physicians as unwell tend to have negative views of their care. Most participants described unwell physicians as less connected within the medical community, and thus limited in making appropriate referrals; less competent and more likely to make errors; less appropriate with patients or staff; disorganized; and more likely to place added responsibilities on patients to limit their problem list and self-diagnose. Participants also described feeling less comfortable with and less trusting of unwell physicians. Feelings of trust and confidence may be threatened to the point where patients seek care elsewhere, particularly if patients feel they are not treated as a whole person and engaged in their own care in a compassionate and empathetic way. Furthermore, participants described questioning and being unwilling to follow the recommendations of unwell physicians; altering their behaviors during appointments such that they limited their discussion with the physician; and even having compassion and concern for unwell doctors whereby they altered their behaviors to prevent overwhelming them. Importantly, however, a few participants explained that a physician’s physical appearance of being unwell may become less important over time as a relationship builds, while other participants explained how unwell physicians may be more relatable since they understand the challenges patients face.

**Table 2:** How patients’ perceptions of physician wellness impact patients’ views of their care, their feelings, and their actions.

Themes	Sub Themes	Description	Sample Quotations ^a^
Their Views of their Care	Doctors’ Connections within Medicine	How capable a doctor appears to be in building working relationships with other providers e.g., whether they are connected to other providers for referrals	*“…generally when people get stressed they withdraw therefore I am less confident that my doctor has access to the appropriate surgeons or dieticians or whatever it is*, *I may need outside of what the doctor could provide because he hasn’t nurtured those connections he you know has withdrawn and so can’t nurture those connections because he doesn’t do anything*.*”* [17]
	Competence	How proficient a doctor appears to be e.g., ability to assess information, thoroughness, making errors	*“I think it’s important that doctors look like they have the capacity to do their job correctly and provide proper patient care*. *If…there’s a doctor that doesn’t look like that because…they’re probably under stress themselves*, *or they’re unkempt then you absolutely have to question whether they’re able to deliver the standard of health care that you need*.*”* [2]
	Appropriateness in Relationships	How appropriate a doctor is when interacting with patients e.g., sexual or abusive, disrespectful, less compassionate	*“…doctors don’t take enough vacations*. *They don’t have hobbies*. *They are so busy with their practices trying to make money that they become irritable and they become um unhealthy to work with*. *I know…a surgeon who is really unpleasant to work with*, *and his bedside manner is terrible*. *And he treats his staff like dirt…When I had to get my knee replacement I said do not send me to Dr*. *X because I need a doctor to give me good care…”* [6]
	Organizational Skills	How efficient and organized a doctor appears to be e.g., cluttered office, disorganized filing, mixing up patient information	*“He had the wrong information*, *he had to go back out and come back in…and when he sat down to talk to us he wasn’t really on top of what*, *what was going on*, *he actually had to apologize about 3 times while we were there for the fact that he didn’t really know what the hell was going on*, *‘cause his record keeping and stuff was so bad*.*”* [3]
	Patient Responsibility	How much accountability a doctor seems to place on the patient e.g., asking patients to prioritize their problems, limit their problem list, or self-diagnose	*“…my visits are very quick and short*, *I’m in and out of the doctor’s office within a minute you know*? *…I tell her what is going on*, *I tell her the tests that I need*, *I tell her basically everything*, *she doesn’t tell me anything to do*, *she doesn’t follow up*, *she doesn’t suggest anything…I am very unhappy with her*.*”* [19]
Their Feelings	Comfort	How comfortable the patient is with the doctor e.g., patient happiness or satisfaction, patients willingness to open up	*“Doctors that look unwell make me uncomfortable in the sense that I look at them in a judgment piece like*, *you’re not taking care of yourself*, *how can you be fully present here with me*? *And I start worrying about them*.*”* [4]
	Trust	How confident the patient is in the doctor e.g., patients may be less confident in an unwell doctor	*“…I rarely doubt the medical advice that they are capable of giving but certainly you know we pick up on so much as people that even if it is conscious or subconscious you may have a lack of or loss of trust*.*”* [10]
	Desire to See the Doctor	How inclined the patient is to continue seeing the doctor e.g., patients may not want to continue seeing an unwell doctor	*“[doctors have a professional responsibility to be well]…it’s their business…You wouldn’t go to a physiotherapist or weight trainer that was overweight or had injuries*. *Or a trainer that sat at their desk and yelled at you wouldn’t be somebody I would want to go see…Makes you second guess what they are doing*, *it would be different if they had or were in a car accident those are certain forms of being unwell but what you take in to be healthy*, *to be well rounded*, *not to be stressed those are things that are in people’s control…And if a person isn’t controlling themselves how are they supposed to help you do that”* [13]
	Being Treated in a Humanistic Manner	Whether the patient feels they are treated as a whole person and engaged in their own care e.g., collaboration between doctor and patient, empathy and compassion, taking an interest in the patient as a person	*“…she just turns and stares at a computer you know*, *a minute into my visit with her she says what do I want and this and I tell her and she says okay and then she just turns and stares into her computer and I am just like what*, *so the first time she did it I didn’t know what to do*, *and then the second time and the third time I guess it’s time for me to go so I just put my coat on and walked out the door*. *She could have asked me do I have any concerns or any questions anything but no she just turns and stares at her computer and types whatever in the new prescription or whatever she puts into her computer but I don’t know just more interaction you know*, *acknowledge me like I am a person I am there*, *I might not understand everything happening you know I guess that I had to find out*, *go online and do my own research and stuff*.*”* [19]
Their Actions	Willingness to Follow Recommendations	How likely the patient is to comply with the doctor’s advice e.g., patients may be less likely to follow advice from doctors who look unwell	*“…if your doctor’s talking to you about obesity and about cholesterol*, *about exercising*, *but it’s very clear and evident this is missing in their own personal life*, *you wonder how important it really is*.*”* [2] *“…I don’t want to take the advice of somebody who I am not sure is doing her job well*.*”* [13]
	Compassion and Concern for the Doctor	How worried a patient may be about the doctor on a personal level e.g., patients worry about doctors who appear unwell	*“…I start worrying about them*, *especially if I’m in a relationship with them over a period of years*, *I’ll send them gifts or cards because you know I worry about them*.*”* [4]
	Altering their Interactions	How the patient conducts him/herself during appointments with the doctor e.g., limiting the number of problems they discuss	*“Maybe they had three things to talk to the doctor about*, *and maybe they will just talk about the most important one and leave the other two for another day because the doctor is stressed out*.*”* [15]

^a^ Numbers indicate the source of the quote based on the participant’s unique identifier.

The third premise is that patients perceive a bi-directional link between physician wellness and patient care (see Table 3). Participants described physicians as human beings with wellness needs who face patient and societal expectations that challenge their abilities to maintain wellness. The participants highlighted the need for physicians to implement the strategies they advise for patients; to role model these behaviors; to set boundaries around their work; to manage stress; and to lead a healthy and balanced life. Furthermore, participants described how physicians’ wellness may be impacted by the care they provide as they work within a challenging healthcare system with high work demands and expectations; poor access to resources for patient care and physician support; and a lack of patient accountability for self-care. Lastly, participants highlighted the cyclical relationship where physician wellness impacts patient care (i.e., unwell doctors put patients at risk) and patient care impacts physician wellness (i.e., putting patients first can limit doctors’ self-care).

**Table 3:** Patients’ beliefs about how doctors are impacted by the care they deliver to their patients.

Themes	Sub Themes	Description	Sample Quotations ^a^
Doctors are Human Too	Patient and Societal Expectations	Social norms about what doctors should be e.g., facing higher standards, stigma, the culture of medicine	*“…in society you know doctors have been sort of placed on a pedestal and viewed as being demi-gods or something you know*, *they are in the end just people and they suffer from the same illnesses and same kind of stress that we do in our lives and aren’t always given the room to look out for themselves*.*”* [12]
	Implementing the Strategies they Advise for Patients	Whether the doctor follows and role models the advice he/she gives to patients e.g., doctors need to maintain their wellness just like patients, preventive medicine, modeling healthy behaviors	*“…one of my favorite quotes is from Mark Twain*. *‘Best chance of success in life is following the advice we give others*.*’ And I think physicians really need to heed that*.*”* [9]
	Managing Stress	How well the doctor copes with pressures e.g., learning how to deal with stress, having an outlet for dealing with stress	*“It’s probably going to be a stressful job*, *but again the ones that looked like they were dealing with it properly somehow*, *that managed to manage their own*, *emotional and physical wellbeing to the point where they looked fit*, *healthy*, *strong*, *capable and caring*.*”* [3]
	Leading a Healthy and Balanced Life	The doctor’s wellness behaviors and practices e.g., exercising, not smoking, having proper nutrition, being well-rounded, maintaining boundaries and balance, knowing when to step back, recognizing their own limits	*“Take a sick day or you know*, *remove themselves from a situation where*, *that they are not in shape in order to deal with*.*”* [12]
Systems Factors	Work Demands and Expectations	What the system and profession require of doctors e.g., long work hours, high patient load, demanding profession with high standards, high stakes situations	*“…I always find that surprising*, *that we put doctors on floors that are 12*, *14*, *16*, *20 hours into shifts and it doesn’t make any sense to me whatsoever*, *so I think those are systematic failures…* [10]
	Resource Availability	How easily the doctor can access resources e.g., doctors need support, resources need to be accessible	*“So if there is something there right now that’s available that’s going to help them take better care of themselves*, *um I’ve read about how psychiatrist end up going to psychiatrists when…going through school and interning*. *I’ve read books about doctors and how they lose a patient and um I don’t know who helps them with that if they receive counseling…there’s a lot more to it than just saying you have a disease let’s give you this medicine and deal with it that way*.*”* [4]
	Patient Self-Responsibility	The patient’s recognition that he/she is accountable for his/her own health e.g., doctors can only do so much, patients need to be accountable for their health	*“…doctors are people too and they have bad days and they have good days and they are just like everybody else their wellness and their mental health can’t be perfect all the time because it’s impossible so we [patients] need to take some ownership for things that happen as well and just*, *you know*, *do some research into things as well…”* [15]
Cyclical Relationship between Physician Wellness and Patient Care	Physician Wellness Impacts Patient Care and Risk	How the doctor’s wellness affects the care that patients receive e.g., unwell doctors may put patients at risk, doctor unwellness impacts patient outcomes and the doctor-patient relationship	*“It’s a bit like when you’re on the airplane and you know*, *the attendant is telling you*, *you have to put your oxygen mask on and then you put the oxygen masks on your children*. *If a doctor is not looking after their own wellness then I don’t think it’s reasonable to expect that they’re delivering the standard of care that the patient may need*, *and certainly the system should be providing*.*”* [2]
	Patient Care Impacts Physician Wellness	How the doctor’s wellness is affected by the care they provide to patients e.g., putting patients first limits doctors’ self-care, emotional demands of the work	*“People walking in with the expectation that doctors are supposed to fix them*. *And doctors being the professionals that they are and having taken an oath to do the best they can I think that must be overwhelming or frustrating or energy sucking for any better term where you go in and it’s a constant mill of ‘help me*, *help me*, *help me*.*’”* [4]

^a^ Numbers indicate the source of the quote based on the participant’s unique identifier.

## Discussion

Our study provides a patient perspective on physician wellness and its links to patient care. Specifically, it suggests that patients observe cues from physicians that are interpreted as signs of wellness or unwellness. Patients then make judgments about a physician’s wellness based on these observations, and these judgments may impact how patients perceive their care, feel about, and act within doctor-patient interactions.

These findings extend the work of Puhl, who documented that physicians viewed by patients as overweight are perceived as less credible and trustworthy than those of normal weight [24]. Our results are also in line with De Vries’ systematic review showing that patients judge clinician empathy as lower if patients perceive staff to be busy and patients remember less information and feel their situation is more dire when presented by a physician who appeared worried [29]. At the same time, recent work suggests that patients may be less comfortable seeing active, fit physicians who share information about their healthy lifestyle, as patients may believe these physicians will judge them for being overweight or having unhealthy habits [30]. This view was shared by some of our participants, suggesting the relationship between physician wellness and doctor-patient interactions is complex.

While we did not examine whether patients’ perceptions of physician wellness match physicians’ self-perceptions or more objective measures, our participants suggested that their interpretations had important consequences. Regardless of more objective measures or physicians’ own perceptions of their wellness, the way in which patients perceive a physician’s wellness can impact how patients experience, feel, and act during the interaction. Halbesleben similarly identified a negative association between patient-observed physician depersonalization and patient satisfaction and recovery time, supporting the idea that patients’ perceptions have real consequences on how they feel about an encounter and their care [11]. Moreover, Halbesleben shows a positive association between patient-observed physician depersonalization and more objective measures of depersonalization, suggesting that patients may accurately assess physician wellness. Others have also found that chaotic work environments and a lack of organization may be related to burnout [31], further suggesting that the cues identified by our participants may be accurate markers of physician wellness.

Another important finding from our study is that patients who perceive a doctor as unwell may alter their actions during doctor-patient interactions. Participants recounted feeling compassion and concern for physicians viewed as unwell, with some participants reportedly altering their behaviors during appointments. Patients described limiting the number of problems they discussed or minimizing symptoms to avoid overwhelming the physician. This reverse caring was also described by Ratanawongsa’s team who found that although physician burnout was not significantly associated with patients’ satisfaction, confidence, or trust (a finding that differs from other studies), burnout was associated with patient-to-physician communication [32]. Patients seeing physicians with greater burnout were more likely to use comforting and optimistic statements compared to those seeing physicians with less burnout. The authors proposed that patients may recognize signs of burnout in their physicians, and in turn, patients may respond to these cues in empathetic and supportive ways.

Furthermore, our results suggest that patients hold a nuanced view of a bi-directional link between physician wellness and patient care. Physician wellness impacts patient care, but physician wellness is also impacted by the care they provide, since physicians are human beings who may face wellness challenges in their work. One such challenge is the medical profession’s complex contract with society which yields expectations and obligations that may impede wellness [33]. In previous work, physicians described feeling awkward when carrying or eating food in patient care settings because they felt patients may view this as unprofessional [23]. These concerns, along with a lack of time and inconvenient access to food, created barriers to adequate nutrition at work. However, our participants highlighted that physicians are not “gods” and that it is unreasonable to expect them to always be well. Rather, there may be a cost to caring for patients and working within the complex healthcare system where physicians may not receive adequate support in caring for patients or themselves. Of interest, Lafreniere found a positive association between residents with higher depersonalization measures and higher patient ratings of residents’ empathy and enablement [34]. Although the authors suggest that these results are somewhat conflicting, the study may further support that caring has a cost for physicians. The empathetic residents suffer burnout, but perhaps maintain their empathy through being more self-critical of their interactions with patients. Linzer also found that time pressures and a chaotic work setting were linked to burnout [35], further suggesting how the healthcare system may harm physicians’ wellness.

Our results should be interpreted within the limitations of the study design. This is an exploratory study intended to generate hypotheses and deepen our understanding. As such, we sampled patients from a single urban center and our sample included a large proportion of retired older adults with relatively frequent interactions with the healthcare system. While our study provides a rich understanding of these patients’ experiences and perceptions, their views may not represent patients more broadly. Similarly, we recruited participants from outpatient settings, and most of their responses appeared to reflect outpatient experiences. Patients’ views of physician wellness presented here may be most reflective of that care setting, and it is possible that patients hold different views about physician wellness in acute care settings where they may have less developed relationships with the physician and where their healthcare needs are more complex or urgent. Lastly, no interviewers were male and this may have influenced participants’ responses.

Our results highlight how patients’ perceptions regarding physician wellness may have important impacts on patient care and the doctor-patient relationship. Patients’ views and judgments about a physician’s wellness may impact patients’ assessments of their care, their feelings of trust, comfort, and holistic treatment, and their actions, such that that they may forego their own needs in order to protect a physician they perceive as unwell. Our participants also suggested that physicians’ work context has an important impact on physician wellness such that physicians may be at risk of being unwell because of their work. While it is possible that patients’ views regarding a physician’s wellness are based largely on observable physical appearance and may not match physicians’ self-perceptions or more objective measures of physician wellness, patients’ perceptions are nevertheless important to doctor-patient interactions and patients’ assessments of their care. This raises the question as to where the added burden of maintaining physician wellness lies—within the clinician’s already heavy-laden job description or within changes to healthcare systems and societal expectations of physicians. Given patients’ awareness of the wellness risks inherent in practicing medicine, patients may be powerful allies in supporting system-level physician wellness initiatives with shared responsibility between individual physicians, the medical profession, and healthcare organizations to ensure physicians are at their best to care for patients. That is, patients may support the idea that the healthcare system and medical profession need to better support physicians, rather than expecting physicians to be solely responsible for their wellness—a view that is becoming increasingly common in the literature on physician wellness and burnout [36–38].

## Supporting information

S1 File Semi-structured interview schedule. (DOCX)
