# Supplementary material for: Understanding how patients perceive physician wellness and its links to patient care: A qualitative study
Source: PLoS One. 2018 May 15;13(5):e0196888. doi: 10.1371/journal.pone.0196888 (PMC5953450; doi:10.1371/journal.pone.0196888)
Supplement: S1 File — (DOCX) [file pone.0196888.s001.docx]

**
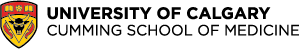
**


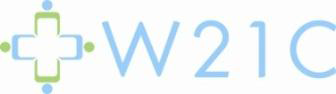

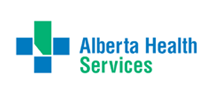


**S1 File. Semi-Structured Interview Schedule.**

**Patients’ Perceptions of Physician Wellness and**

**Its Links to Patient Care: A Qualitative Study**

**Preamble for patients:**

*Our research team is exploring what people think about doctors’ wellness. We’d like to talk to you today about your thoughts on the health and well-being of doctors and whether or not it links to how they care for patients. Please feel free to answer our questions based on your general ideas about this topic, or by thinking of examples from your own health care experiences, or those of family and friends.*

**Semi-structured interview questions:**

1. What do the terms “doctor wellness”, “doctor health” or “doctor well-being” mean to you?
2. From your perspective, what might doctor health, well-being or wellness look like?
   1. Further probes: What about their physical health/wellness, mental, psychological or emotional health/wellness?
3. From your perspective, what might the opposite look like, for example unwell doctors?
   1. Further probes: Doctors who are physically unwell, or mentally, psychologically or emotionally unwell?

1. Does that idea of wellness in doctors differ from how you see wellness in yourself and in your own life? If so, what are some of the factors that you think make health or wellness different for doctors?

1. Do you think that a doctor’s wellness is linked to patient care? If so, how do you think it is linked? Can you give us some examples?
   1. Further probes: Is it linked to their ability to provide care? Does it impact the physician-patient relationship?
2. Earlier we asked you what a healthy or well doctor might look like. Do you think that a doctor’s appearance of being well or unwell could influence their relationship with the patient? If so, can you give us some examples of how that might happen?
3. Do you think doctors have a professional responsibility to be well? If so, what do you think doctors should do to stay well?

1. Is there anything else you would like to tell us about your thoughts on physician wellness, or about its links to patient care?

**Background Information**

Now I need to ask you some questions about your personal and work characteristics. Your answers to these questions will again be confidential, and will help us interpret your responses and compare them with others. Your answers from the interview questions will be presented in such a way that you cannot be identified by your responses or the personal and work characteristics that I’m going to ask you about next.

- In what year were you born? 19_____
- Gender? __________
- Which of the following best describes your present marital situation?

□ Single/never married □ Co-habitating/common law □ Married

□ Separated □ Divorced □ Widowed

If you have a partner, how many years have you been married? ________

- How many children do you have?________________
- How many children are currently living at home with you and what are their ages?

_______ child/ren under 6 years of age _______ child/ren 13 to 18 years of age

_______ child/ren 6 to 12 years of age _______ child/ren 18 years of age or older

- What is your current occupation? ____________________________
- What level of education have you completed?

_____high school _____college or university degree

- Compared with other people your age, how would you describe your physical health?

□ Poor □ Fair □ Good □ Very good □ Excellent

- Compared with other people your age, how would you describe your mental or emotional health?

□ Poor □ Fair □ Good □ Very good □ Excellent

- How often do you have contact with the health care system?

□ Less than once a year □ Once or twice a year □ Every 2-3 months □ Almost every month

IN CLOSING

**Thank you for your input and participation in this project. Your time and insights are greatly appreciated.**

**Do you have any other comments you’d like to add in light of what we’ve been talking about?**

**Was there anything you were expecting us to ask that we’ve missed?**

**If you think of anything else you’d like to add, please feel free to send me a note or email.**
